# Reducing interaction in simultaneous paired stimulation with CI

**DOI:** 10.1371/journal.pone.0171071

**Published:** 2017-02-09

**Authors:** Dirk Vellinga, Saskia Bruijn, Jeroen J. Briaire, Randy K. Kalkman, Johan H. M. Frijns

**Affiliations:** ENT-Department, Leiden University Medical Centre, Leiden, The Netherlands; University of California Irvine, UNITED STATES

## Abstract

In this study simultaneous paired stimulation of electrodes in cochlear implants is investigated by psychophysical experiments in 8 post-lingually deaf subjects (and one extra subject who only participated in part of the experiments). Simultaneous and sequential monopolar stimulation modes are used as references and are compared to channel interaction compensation, partial tripolar stimulation and a novel sequential stimulation strategy named phased array compensation. Psychophysical experiments are performed to investigate both the loudness integration during paired stimulation at the main electrodes as well as the interaction with the electrode contact located halfway between the stimulating pair. The study shows that simultaneous monopolar stimulation has more loudness integration on the main electrodes and more interaction in between the electrodes than sequential stimulation. Channel interaction compensation works to reduce the loudness integration at the main electrodes, but does not reduce the interaction in between the electrodes caused by paired stimulation. Partial tripolar stimulation uses much more current to reach the needed loudness, but shows the same interaction in between the electrodes as sequential monopolar stimulation. In phased array compensation we have used the individual impedance matrix of each subject to calculate the current needed on each electrode to exactly match the stimulation voltage along the array to that of sequential stimulation. The results show that the interaction in between the electrodes is the same as monopolar stimulation. The strategy uses less current than partial tripolar stimulation, but more than monopolar stimulation. In conclusion, the paper shows that paired stimulation is possible if the interaction is compensated.

## 1. Introduction

Cochlear implants (CIs) are widely used in profoundly or severely hearing impaired patients. The device works with an electrode array, which is placed inside the cochlea. From this electrode array, the cochlear nerve is directly stimulated by small electric pulses. Due to the tonotopic organization of the cochlea, each electrode contact in the array induces a different pitch percept. This, combined with a speech coding strategy, enables the patient to perceive sounds and, in most cases, understand speech.

The most commonly used speech coding strategies in modern CIs are variations on continuous interleaved sampling (CIS) [1] in which the speech signal is divided into frequency bands, which are then stimulated sequentially, one directly after the other, to prevent electrical interaction between the electrodes. All current CI brands have their own variations on this principle [2, 3]. It can, however, be beneficial to stimulate pairs of electrodes instead of single electrodes. Paired stimulation can be useful for two reasons. Firstly, it can double the stimulus rate per electrode while keeping the phase duration equal. In theory, a higher stimulation rate per channel can be beneficial for speech understanding, because the timing of the speech queues can be delivered more accurately [4]. However, there are also studies that contradict this theory, for example [5]. Still it might be beneficial for individual patients to increase the stimulation rate. Alternatively, with paired stimulation the overall stimulation rate can be halved while keeping the stimulation rate per channel the same. If the overall stimulation rate is reduced, longer pulses can be used, which reduces the voltage needed for stimulation. If the battery voltage can be reduced, the power consumption can be lower, which leads to increased battery life.

However, the studies of Buechner et al. [6] and Bonnet et al. [7] have shown that paired stimulation leads to lower speech recognition than CIS. This is most likely caused by increased interaction between the electrodes. Several methods to reduce interaction have been proposed in the past[8]. Zierhofer and Schatzer [9] have suggested channel interaction compensation (CIC) as a method to reduce the interaction between electrodes with multi-channel stimulation. Their method is based on compensating the interaction on the stimulation electrodes based on the individual impedance matrix of the patient. Another method to reduce the interaction during paired stimulation was suggested by Shefin et al. [10]. This study, performed on cats, shows that the interaction during paired stimulation can be reduced by phased array stimulation. Phased array stimulation uses the impedances between electrodes, measured for each patient separately, to create a stimulus which is zero on all electrodes except for the stimulating electrode [11–15].

The aim of this study is to investigate some promising methods to compensate for the interaction between the electrodes during paired stimulation in humans. Psychophysical experiments are used to compare the interaction and loudness growth for paired stimulation in several previously proposed stimulation methods and a novel approach. We will use single monopolar stimulation (sMP) and uncompensated paired monopolar stimulation (pMP) as references and compare these with CIC [9], partial tripolar stimulation (pTP) [14–17] and a novel approach that we will call phased array compensation (PAC).

### 1.1 Paired stimulation strategies

**Fig 1:**
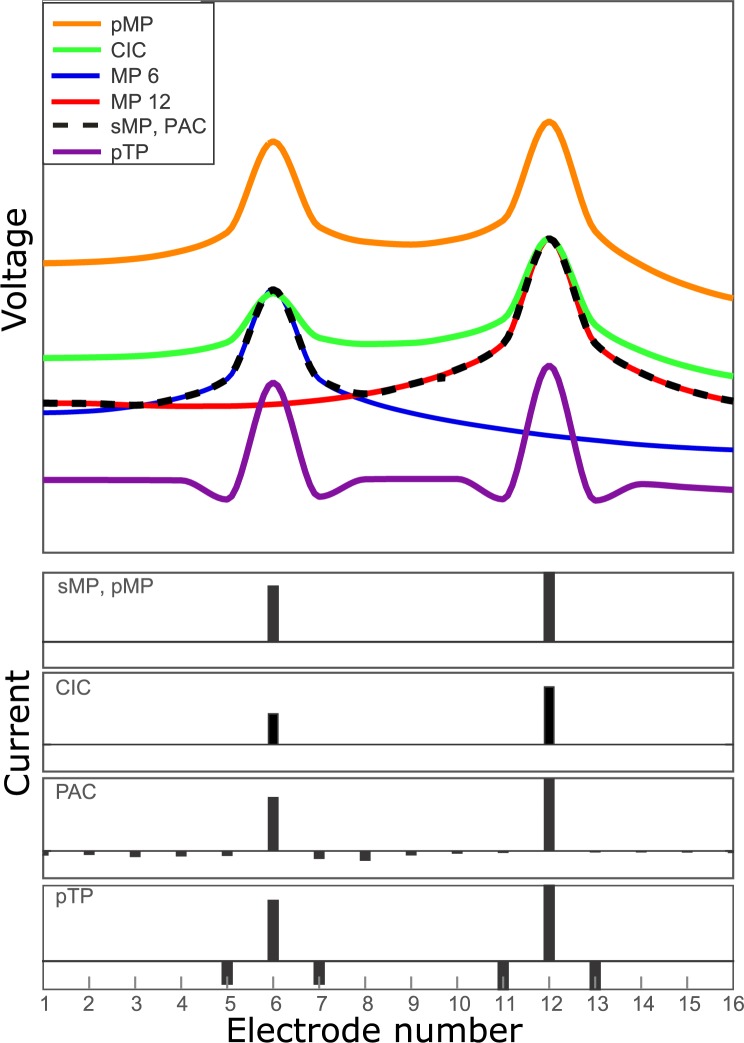
Simulated potential along the array and currents per electrode of the CI for the different stimulation strategies. The current graphs on the bottom of the figure represent the currents from which the potentials are calculated for each stimulation method. The currents are based on the same current vector for each stimulation method. Then the currents are compensated with the compensation methods to acquire the shown data.

The different paired stimulation strategies are illustrated in Fig 1. The curves show the calculated potential along the cochlea for the different stimulation strategies. The curves are interpolated from simulated electrode potentials in a realistic three-dimensional volume conduction model of the implanted human cochlea, described by Kalkman et al. [18, 19]. To compare the effects of the different stimulation strategies the curves in this figure are not loudness compensated. Instead, a standard current on two electrodes is chosen as an example and then the compensation algorithm for each of the compensation methods is used to obtain the currents on the bottom of the graph. From these currents the voltage for each stimulation method is calculated. As a reference we used sMP and pMP stimulation strategies. The individual stimulations of electrode 6 (blue) and electrode 12 (red) are shown in Fig 1 as references. With sMP we presume the channels to be completely independent (i.e., no carry-over effects from the first pulse to the second one). The total potential in the cochlea would therefore be described by the contour potential of the two individual electrode stimulations (black dotted line). When we stimulate the electrodes in pairs, however, the potentials of the monopoles add up and we see from the orange line in Fig 1 that the resulting potential in the cochlea is much higher due to interaction between the two stimulating electrodes.

In this paper we use three different compensation strategies, with the intent to reduce the adverse effects of interaction between the electrodes. First of all, we investigate the effects of CIC, which has been suggested as a multi-channel stimulation strategy by Zierhofer and Schatzer [9]. The idea of this stimulation strategy is to reduce the current on the stimulated electrodes during multi-channel stimulation in such a way that the stimulating potential on the electrodes themselves is the same as in the CIS strategy, which intends to correct for the interaction between the electrodes. This is accomplished by taking into account the measured impedance matrix of the patient. However, it does not compensate for the increased potential between the electrodes. The principle is illustrated in Fig 1 (green line). On the contacts the current is equal to the individual monopolar stimulations, but between the contacts there is still an elevated potential compared to the monopolar stimulation strategy due to increased interaction. The method eliminates the loudness effect of paired stimulation, but does not reduce the interaction in between the electrodes [9].

Besides CIC we also investigated pTP stimulation as a method to reduce the interaction during paired stimulation. This method uses a negative current on the electrodes adjacent to the main electrode. The compensating current on the side electrodes can be described as -0.5 σ I_m_ in which σ is the tripolar compensation coefficient and I_m_ is the current on the center electrode. For this study we have chosen σ = 0.75, based on the research of Litvak et al [17] and Vellinga et al [8]. This leads to a decreased interaction between the electrodes, as can be seen in Fig 1. However it also causes a decrease of the potential on the main electrodes, leading to less loudness. This means that I_m_ has to be increased in order to reach the same loudness as with monopolar stimulation.

Lastly, in this paper we investigate a novel approach to paired stimulation called phased array compensation (PAC), which is based on phased array stimulation [11–13, 20] and CIC [9]. This method uses all electrodes in the array to get exactly the same potential along the array as with two sequential monopolar stimulations. In CIC unstimulated electrodes and electrodes which have a negative current amplitude (or different phase) are excluded from stimulation. In PAC, all electrodes are used to get a stimulating potential, which is as close as possible to the two sequentially stimulated monopolar stimulations. This makes it possible to not only get the desired potential on the stimulating electrodes, but also on the intermediate contacts. This is depicted in Fig 1 with the dotted black line.

## 2. Materials and methods

### 2.1 Subjects

Nine (from which eight were able to complete all experiments) post-lingually deaf patients with an Advanced Bionics (AB) HiRes90K Hifocus 1J implant with 16 electrodes and at least one year of CI experience are selected for this study. The selected subjects are relatively good performers with a minimal phoneme score of 75% at 65 dB SPL on a monosyllabic consonant, vowel, consonant (CVC) words test. In our clinical population about 70% of our post-lingually deaf patients meet this criterion (results will be published elsewhere). The subjects’ demographics are listed in Table 1. Subject S03 (who was our original tenth subject) was unable to finish the experiment due to facial nerve stimulation occurring before reaching the loudness required for the experiment with the electrodes around electrode 6–7, in all strategies. In his clinical program, this subject had reduced M-levels in these regions as well due to facial nerve stimulation. Subject S09 stopped after the loudness experiment because of reasons unrelated to the experiment. Permission from the Medical Ethical Committee of the LUMC was obtained under number P02.106. All subjects have given their written and oral consent to participate in the experiments. This procedure and the consent form was approved by the Ethical Committee.

**Table 1:** Clinical and biographical data on the subjects included in this study.

Subject	Sex	Age (yr)	Duration of deafness (yr)	Implant experience (yr)	Phoneme score CVC test (%)*	Etiology of hearing loss
S01	f	57	17	1	90	Usher syndrome
S02	f	40	2	2	95	Meningitis
S03	m	67	13	1	99	m. Meniere
S04	m	69	8	3	78	m. Meniere
S05	f	47	12	3	77	Familial progressive
S06	m	22	3	2	85	Enlarged vestibular aqueduct
S07	f	71	16	2	88	Unknown
S08	f	54	31	2	73	Unknown
S09	m	38	2	1	96	Unknown
S10	f	51	6	1	80	Unknown

*The last phoneme scores for CVC words score at 65 dB SPL scored by the patients during a regular clinical checkup.

### 2.2 Software

Experiments are performed with the research tools EFIM (Electric field imaging and modeling, Advanced Bionics LLC) for the impedance measurements, BEDCS (Bionic Ear Data Collection System, Advanced Bionic LLC) for the electrical stimulation and PACTS (PsychoACoustic Test Suite) for the psychophysical testing procedures. Statistical analysis is done with IBM SPSS Statistics (version 20).

### 2.3 Stimuli

Charge-balanced biphasic current pulses at a pulse rate of 800 Hz per electrode and a phase duration of 75 μs are used for all stimuli. Two pairs of electrodes with different electrode distances (3 and 5 contacts separation) are investigated, in order to investigate the effect of distance between the electrodes on the interaction effects. Both pairs are centered around electrode 9 on the implant (numbering follows the manufacturer’s convention, 1–16 from apex to base). This results in the electrode pair of contact 6 and 12 (pair 5) and the contacts 7 and 11 (pair 3). The exception to this stimulus definition are the phase durations in the interaction experiments for subjects S02, S06 and S08. Due to unwanted changes in the experimental software, perhaps caused by the fact that the laptop was used for several experiments, the phase duration was accidentally set to 200 μs for these experiments. To check for possible deviations in the outcomes introduced by the protocol deviation we have normalized the data to the sMP value of pair 5 and performed a mixed model statistical analysis to compare the data, which shows that there is no significant difference between the sets with different pulse widths (p = 0.26).

**Fig 2:**
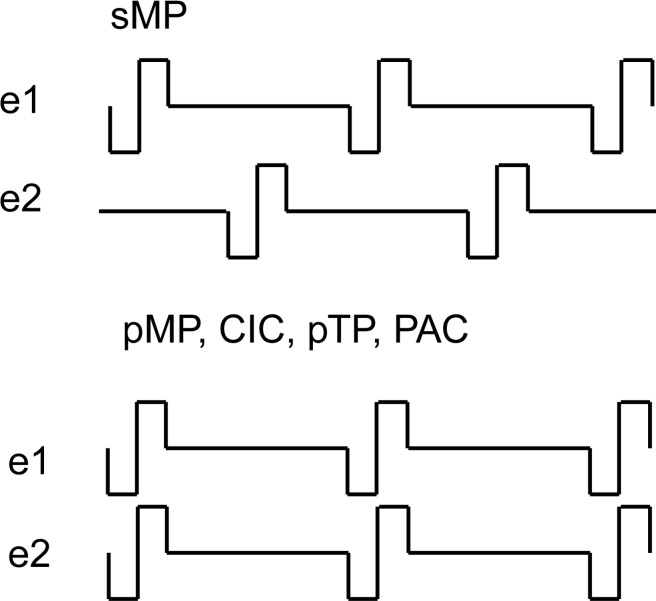
Schematic representation of a small part of the pulse train of two electrodes during sMP (sequential) and pMP, CIC, pTP and PAC (paired) stimulation. During pTP and PAC stimulation more than two electrodes are used at the same time.

A small part of the pulse train of both the sequential and the paired stimulation strategies are depicted in Fig 2. During sMP stimulation, the pulses of the second electrode are timed exactly between the pulses of the first one. During all others, namely pMP, CIC, pTP and PAC, the pulses are all timed exactly simultaneously on all electrodes.The CIC stimuli are calculated exactly according to the paper of Zierhofer et al. [9]. The PAC stimulation is a combination of this stimulation strategy and the phased array strategy suggested by van der Honert et al. [13].

For the PAC stimulation first the current profile of the stimulus has to be calculated, for each patient individually. During the experiments the impedance between each electrode was measured using the EFIM software package, which measures a matrix (Z) of impedances (Z_ij_) between the stimulating electrode (i) and the measuring electrode (j) for each combination of electrodes as described in [21].

$$Z=\left[[\begin{array}{ccc} z_{11} & \cdots & z_{1,16}\\ \vdots & \ddots & \vdots \\ z_{16,1} & \cdots & z_{16,16} \end{array}]\right].$$ (1)

The measured impedances on the diagonal are not reliable [13] because the stimulating and measuring electrode are the same in these cases. These values have therefore been interpolated using the maximum of the linear and exponential fit of the impedances from the neighbours from both sides. This method has been shown to give the best result according to the computer model of Kalkman et al. [18, 19].

With this impedance matrix the voltages at each electrode contact along the array can be calculated by multiplying it with the current vector I of the induced current:

V=Z∙I (2)

The goal of the PAC stimulation is to get the same voltage across the array as would be the case with sMP stimulation. With Eq (2) the voltage vector for the two stimulating electrodes can be calculated separately. The maximum of these two voltage vectors (V_m_) is the desired voltage in PAC stimulation. This is illustrated in Fig 1, the red and the green line are the monopolar stimulations and the covering dotted line depicts the desired voltage during PAC stimulation. The current vector I_PAC_ required to achieve V_m_ can then be calculated by multiplying it by the inverse of Z:

$$I_{PAC}=Z^{inv}∙V_{m}.$$ (3)

### 2.4 Loudness buildup

In this study paired stimulation with equal loudness at the two channels is used. This requires the loudness of the two channels to be balanced for each pair at the most comfortable loudness (MCL). The MCL level for the first channel of the pair (number 6 or 7) was determined by adjusting the loudness until the subject judges the sound to be at MCL level. The MCL level for the second electrode of the pair is determined by loudness balancing it with the first. This is done by keeping the loudness of the first channel at a constant level while the subjects varied the loudness of the second with the arrow keys on the experimental laptop until they considered the levels to be of equal loudness. The stimulation of the first and the second electrode are alternated with 0.5 s intervals stimulations. Coloured squares on the computer screen indicating which stimulus is given. The balancing experiment is performed twice. One time from a higher current and one time from a lower current. The result is averaged.

To determine the current needed to reach a certain loudness, loudness buildup experiments are done for each stimulation strategy and both pairs. This is studied with the 8 point loudness buildup scale from Potts et al [22], ranging from the threshold level (1) to upper limit of comfortable loudness (8). During the experiment the experimenter increases the current on the electrodes gradually. When the subjects first hear a sound, this is considered the threshold level. The subject is asked to indicate when the loudness reaches the next step on the scale, the current level is then again noted down. The stimulation was stopped immediately when level 8 is reached. The experiment is repeated three times for each strategy and each subject.

### 2.5 Interaction

To investigate the interaction of the paired stimulation a forward masking experiment on electrode 9 (exactly in the middle of both electrode pairs) is performed. Before the experiment all the pairs are balanced at MCL level. The same procedure as described in paragraph 2.4 is used, the sMP strategy for pair 5 is chosen as a reference for all balancing experiments. The balancing experiment is repeated four times, twice from a higher loudness level and twice from a lower loudness level. The final result is the average of the four experiments.

For the interaction we use the psychophysical forward masking paradigm also used by Cohen et al. [23]. The masker is a 300 ms pulse train at MCL level for one of the paired stimulations strategies. The probe consists of a monopolar stimulation on electrode 9, which is 5 ms after the masker and has a duration of 20 ms. The threshold of the probe is determined by a three alternative forced choice experiment with a one up, two down paradigm. All paired stimulation strategies are tested in randomized order and are repeated three times per subject. The threshold for the interaction is normalized in each subject by the threshold of sMP of pair 5, which is used as a reference.

## 3. Results

### 3.1 Loudness growth

**Fig 3:**
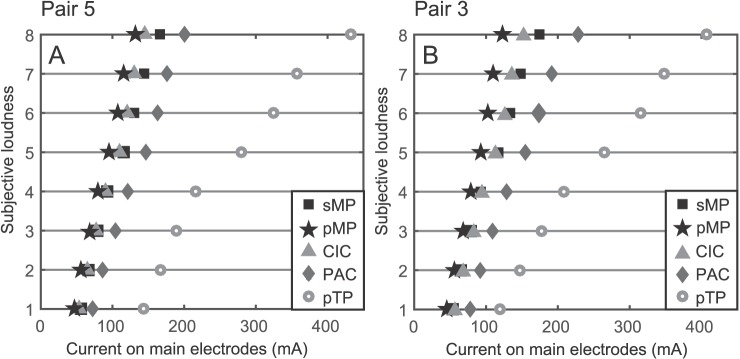
The subjective loudness is plotted against the largest current on the array, averaged over 9 subjects. On the left (A) this is done for pair 5 and on the right (B) for pair 3.

In Fig 3 the loudness growth is plotted for each stimulation strategy for both pairs. The depicted current is the maximum current in the array (which is one of the two main electrodes), averaged over 9 patients. The order of increasing current consumption for the loudness growth is the same for both pairs, namely: pMP, CIC, sMP, PAC and pTP. Most notably, the pTP uses considerably more current than other strategies. Statistical analysis, using a linear mixed effect model with Ŝidák correction for multiple testing, shows that all loudness curves except CIC are significantly different (p<0.01) from sMP, for both pair 3 and pair 5. If they are plotted in decibels, with 0 decibels being the threshold, all curves are linear and overlapping, with a slope of 0.7 ± 0.1 loudness steps/dB, showing that the relative loudness growth is the same for all strategies.

### 3.2 Interaction

**Fig 4:**
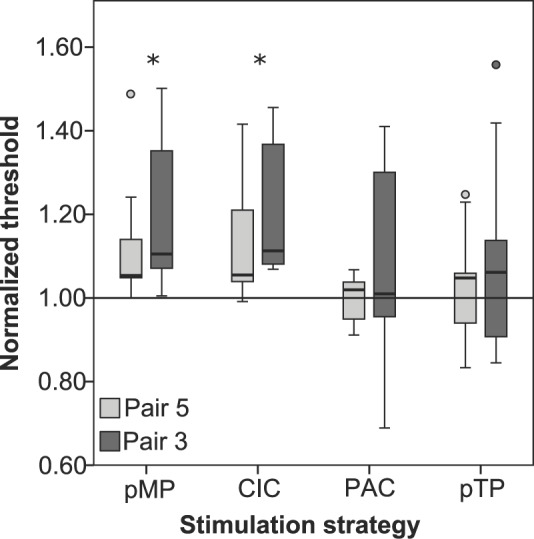
The threshold of hearing at electrode number 9 for each stimulation strategy, normalized to the sMP stimulation strategy, for both pair 3 (dark grey) and pair 5 (light grey). The asterisk indicates a significant deviation from the sMP paradigm (p<0.01). A total of 8 subjects participated in this experiment. The whiskers indicate the minimum and maximum value, the boxes the upper and lower quartile and the filled circles are outliers.

Fig 4 shows the results of the interaction experiment. In the graph the thresholds of probe electrode 9 are normalized to the threshold of the sMP strategy (by dividing the average threshold of the paired stimulation strategy by the average threshold of the sMP strategy). This means that a normalized threshold of 1 indicates a result equal to the sMP strategy, a result above 1 indicates more interaction than the sMP strategy and a result lower than 1 means less interaction than the sMP strategy. The results were analyzed using a statistical mixed model with Ŝidák correction for multiple testing. The pMP and CIC stimulation strategies both show significantly more interaction (p = 0.007 for pMP and p = 0.008 for CIC) than the sMP strategy. The interaction for the PAC (p = 1.000) and pTP (p = 0.998) strategies, however, are equal to the sMP strategy. There is no significant difference in interaction between pair 3 and pair 5 (p = 0.911). Compared to the sMP strategy the probe-only condition (the threshold of electrode 9, without any masker) has a factor 0.6 ± 0.1 lower threshold.

## 4. Discussion

The presented data shows clearly that there is more interaction in uncompensated paired stimulation than sequential paired stimulation. If we compare the loudness growth of paired and sequential stimulation we see that uncompensated paired stimulation needs less current to reach the same subjective loudness level. This can be explained by loudness integration between the two electrodes, as was predicted in the model calculations as shown in Fig 1. The figure shows that when the two potentials are added up (which is the case in uncompensated paired stimulation), the total stimulation voltage is much higher than the voltage of the individual stimulations, which increases the loudness for the same stimulation current. Moreover, the interaction experiments (where the loudness percept is the same, due to balancing) show significantly more interaction in between the stimulating electrodes (at electrode 9) in paired stimulation compared to sequential stimulation.

CIC has been suggested as a method to reduce the adverse effects of interaction on the electrodes while using the CI in paired mode [9]. The data from this paper shows that CIC is indeed very effective in decreasing the loudness integration on the stimulating electrodes since there is no significant difference in loudness buildup between sMP stimulation and CIC. However, the method does not counteract the interaction in between the electrodes. The results from these interaction experiments clearly show that the interaction is significantly increased with CIC stimulation. This is also illustrated in Fig 1, where we see that the voltage in the model simulations from sMP and CIC are the same on the stimulating electrodes, but the voltage of CIC is higher in between them, which has also been predicted in the paper of Zierhofer and Schatzer [9].

Theoretically, the interaction between the electrodes can be reduced by using pTP stimulation. The loudness buildup results show that for pTP much more current is needed to reach the same loudness. This has already been seen in other studies [8, 14, 16, 17]. When the loudness buildup is plotted in a logarithmic scale (with the threshold at 0 dB), however, the loudness growth is the same for all strategies, which has also been shown in the aforementioned studies. The interaction results show that the interaction in pTP is reduced compared to uncompensated paired stimulation and is the same as sequential stimulation. This shows that pTP is effective in counteracting the interaction of paired stimulation, but does so at the cost of a high power consumption.

Finally, we have studied PAC as a novel method to reduce interaction during paired stimulation. With this method we need more current to reach a given loudness level than with sMP stimulation. This increased current requirement is most likely partly caused by errors in the estimation of the diagonal elements in the matrix of Eq 1. An under- or overestimation of the diagonal elements could lead to an error in the compensation current calculations and therefore to an error in the absolute loudness scaling. This could be corrected in the future by an improved model or an improved measurement technique for the diagonal elements of the Z matrix. Despite this disadvantage, the interaction between the electrodes is the same for sequential stimulation in this method, which in our opinion makes it a suitable method for paired stimulation.

## 5. Conclusion

From the data we can conclude that uncompensated paired stimulation increases the interaction in between the electrodes. This has previously been shown to adversely affect speech intelligibility. The paper also shows that this can be counteracted by using either pTP or PAC stimulation instead of monopoles. Using these methods the interaction in between the two stimulating electrodes is the same as when using monopoles. Clinical trials of speech perception with PAC and pTP-based strategies, testing whether this reduced interaction also leads to improved speech perception, are currently underway.
